# Supplementary material for: Baseline Characterization of the Gut Microbiota of Field and Colony Populations of Phlebotomus tobbi and Preliminary Assessment of the Anti-Leishmanial Activity of Cultivable Bacteria
Source: Pathogens. 2026 Jun 23;15(7):658. doi: 10.3390/pathogens15070658 (PMC13416292; doi:10.3390/pathogens15070658)
Supplement: Supplementary file 1 [file pathogens-15-00658-s001.zip › pathogens-4357168-File S1/Composition_Barplots/Figures/charts/CLlOTA2u1RbzHD0kZMACsShdHcrDha_legend.pdf]

k__Bacteria;p__Actinobacteria;c__Actinobacteria;o__Actinopolysporales;f__Actinopolysporaceae;g__Actinopolyspora;s__sp4826
k__Bacteria;p__Actinobacteria;c__Actinobacteria;o__Corynebacteriales;f__Corynebacteriaceae;g__Corynebacterium;s__freneyi-xerosis
k__Bacteria;p__Actinobacteria;c__Actinobacteria;o__Corynebacteriales;f__Mycobacteriaceae;g__Mycobacterium;s__NA
k__Bacteria;p__Actinobacteria;c__Actinobacteria;o__Corynebacteriales;f__Nocardiaceae;g__Rhodococcus;s__rhodochrous
k__Bacteria;p__Actinobacteria;c__Actinobacteria;o__Micrococcales;f__Microbacteriaceae;g__Microbacterium;s__maritypicum-oxydans
k__Bacteria;p__Actinobacteria;c__Actinobacteria;o__Propionibacteriales;f__Propionibacteriaceae;g__Propionibacterium;s__acnes
k__Bacteria;p__Actinobacteria;c__Actinobacteria;o__Pseudonocardiales;f__Pseudonocardiaceae;g__Saccharopolyspora;s__sp8119
k__Bacteria;p__Firmicutes;c__Bacilli;o__Bacillales;f__Staphylococcaceae;g__Staphylococcus;s__NA
k__Bacteria;p__Firmicutes;c__Bacilli;o__Bacillales;f__Staphylococcaceae;g__Staphylococcus;s__epidermidis-hominis
k__Bacteria;p__Firmicutes;c__Bacilli;o__Bacillales;f__Staphylococcaceae;g__Staphylococcus;s__saprophyticus-xylosus
k__Bacteria;p__Firmicutes;c__Bacilli;o__Bacillales;f__Staphylococcaceae;g__Staphylococcus;s__succinus
k__Bacteria;p__Firmicutes;c__Bacilli;o__Lactobacillales;f__Enterococcaceae;g__Enterococcus;s__casseliflavus-flavescens-gallinarum
k__Bacteria;p__Firmicutes;c__Bacilli;o__Lactobacillales;f__Enterococcaceae;g__Enterococcus;s__faecalis
k__Bacteria;p__Firmicutes;c__Bacilli;o__Lactobacillales;f__Leuconostocaceae;g__Fructobacillus;s__tropaeoli
k__Bacteria;p__Firmicutes;c__Clostridia;o__Clostridiales;f__Lachnospiraceae;g__Anaerostipes;s__hadrus
k__Bacteria;p__Firmicutes;c__Clostridia;o__Clostridiales;f__Lachnospiraceae;g__Blautia;s__wexlerae
k__Bacteria;p__Firmicutes;c__Clostridia;o__Clostridiales;f__Ruminococcaceae;g__Faecalibacterium;s__sp34558
k__Bacteria;p__Proteobacteria;c__Alphaproteobacteria;o__Rhizobiales;f__Bradyrhizobiaceae;g__Afipia-Bradyrhizobium;s__NA
k__Bacteria;p__Proteobacteria;c__Alphaproteobacteria;o__Rhizobiales;f__Bradyrhizobiaceae;g__NA;s__sp43413
k__Bacteria;p__Proteobacteria;c__Alphaproteobacteria;o__Rhizobiales;f__Brucellaceae;g__Brucella-Ochrobactrum;s__NA
k__Bacteria;p__Proteobacteria;c__Alphaproteobacteria;o__Rhizobiales;f__Methylobacteriaceae;g__Methylobacterium;s__radiotolerans
k__Bacteria;p__Proteobacteria;c__Alphaproteobacteria;o__Rhizobiales;f__NA;g__Phreatobacter;s__oligotrophus
k__Bacteria;p__Proteobacteria;c__Alphaproteobacteria;o__Rhodospirillales;f__Acetobacteraceae;g__Asaia;s__NA
k__Bacteria;p__Proteobacteria;c__Alphaproteobacteria;o__Rhodospirillales;f__Acetobacteraceae;g__Asaia;s__sp45567
k__Bacteria;p__Proteobacteria;c__Alphaproteobacteria;o__Rickettsiales;f__Rickettsiaceae;g__Rickettsia;s__NA
k__Bacteria;p__Proteobacteria;c__Alphaproteobacteria;o__Sphingomonadales;f__Sphingomonadaceae;g__Sphingomonas;s__NA
k__Bacteria;p__Proteobacteria;c__Alphaproteobacteria;o__Sphingomonadales;f__Sphingomonadaceae;g__Sphingomonas;s__adhaesiva-ginsenosidimutans
k__Bacteria;p__Proteobacteria;c__Alphaproteobacteria;o__Sphingomonadales;f__Sphingomonadaceae;g__Sphingomonas;s__alpina-echinoides-glacialis
k__Bacteria;p__Proteobacteria;c__Alphaproteobacteria;o__Sphingomonadales;f__Sphingomonadaceae;g__Sphingomonas;s__aquatilis-hunanensis-melonis
k__Bacteria;p__Proteobacteria;c__Alphaproteobacteria;o__Sphingomonadales;f__Sphingomonadaceae;g__Sphingomonas;s__desiccabilis
k__Bacteria;p__Proteobacteria;c__Alphaproteobacteria;o__Sphingomonadales;f__Sphingomonadaceae;g__Sphingomonas;s__parapaucimobilis
k__Bacteria;p__Proteobacteria;c__Betaproteobacteria;o__Burkholderiales;f__Alcaligenaceae;g__Achromobacter;s__xylosoxidans
k__Bacteria;p__Proteobacteria;c__Betaproteobacteria;o__Burkholderiales;f__Burkholderiaceae;g__Burkholderia-Paraburkholderia;s__fungorum
k__Bacteria;p__Proteobacteria;c__Betaproteobacteria;o__Burkholderiales;f__Burkholderiaceae;g__Ralstonia;s__pickettii
k__Bacteria;p__Proteobacteria;c__Betaproteobacteria;o__Burkholderiales;f__Comamonadaceae;g__Delftia;s__acidovorans
k__Bacteria;p__Proteobacteria;c__Betaproteobacteria;o__Burkholderiales;f__Comamonadaceae;g__Delftia;s__lacustris-tsuruhatensis
k__Bacteria;p__Proteobacteria;c__Gammaproteobacteria;o__Enterobacteriales;f__Enterobacteriaceae;g__Enterobacter;s__NA
k__Bacteria;p__Proteobacteria;c__Gammaproteobacteria;o__Enterobacteriales;f__Enterobacteriaceae;g__Enterobacter;s__cloacae-sp58653
k__Bacteria;p__Proteobacteria;c__Gammaproteobacteria;o__Enterobacteriales;f__Enterobacteriaceae;g__Erwinia;s__aphidicola-persicina
k__Bacteria;p__Proteobacteria;c__Gammaproteobacteria;o__Enterobacteriales;f__Enterobacteriaceae;g__Erwinia;s__persicina
k__Bacteria;p__Proteobacteria;c__Gammaproteobacteria;o__Enterobacteriales;f__Enterobacteriaceae;g__Klebsiella;s__pneumoniae
k__Bacteria;p__Proteobacteria;c__Gammaproteobacteria;o__Enterobacteriales;f__Enterobacteriaceae;g__Morganella;s__morganii
k__Bacteria;p__Proteobacteria;c__Gammaproteobacteria;o__Enterobacteriales;f__Enterobacteriaceae;g__Pantoea;s__agglomerans
k__Bacteria;p__Proteobacteria;c__Gammaproteobacteria;o__Enterobacteriales;f__Enterobacteriaceae;g__Pantoea;s__agglomerans-eucrina
k__Bacteria;p__Proteobacteria;c__Gammaproteobacteria;o__Enterobacteriales;f__Enterobacteriaceae;g__Pantoea;s__ananatis
k__Bacteria;p__Proteobacteria;c__Gammaproteobacteria;o__Enterobacteriales;f__Enterobacteriaceae;g__Pantoea;s__vagans
k__Bacteria;p__Proteobacteria;c__Gammaproteobacteria;o__Enterobacteriales;f__Enterobacteriaceae;g__Serratia;s__grimesii-liquefaciens-proteamaculans
k__Bacteria;p__Proteobacteria;c__Gammaproteobacteria;o__Enterobacteriales;f__Enterobacteriaceae;g__Serratia;s__liquefaciens-myotis-proteamaculans
k__Bacteria;p__Proteobacteria;c__Gammaproteobacteria;o__Enterobacteriales;f__Enterobacteriaceae;g__Serratia;s__marcescens
k__Bacteria;p__Proteobacteria;c__Gammaproteobacteria;o__Enterobacteriales;f__Enterobacteriaceae;g__Serratia;s__marcescens-nematodiphila
k__Bacteria;p__Proteobacteria;c__Gammaproteobacteria;o__Enterobacteriales;f__Enterobacteriaceae;g__Serratia;s__ureilytica
k__Bacteria;p__Proteobacteria;c__Gammaproteobacteria;o__Oceanospirillales;f__Halomonadaceae;g__Zymobacter;s__palmae
k__Bacteria;p__Proteobacteria;c__Gammaproteobacteria;o__Pseudomonadales;f__Moraxellaceae;g__Acinetobacter;s__baumannii-calcoaceticus
k__Bacteria;p__Proteobacteria;c__Gammaproteobacteria;o__Pseudomonadales;f__Moraxellaceae;g__Acinetobacter;s__bouvetii-johnsonii
k__Bacteria;p__Proteobacteria;c__Gammaproteobacteria;o__Pseudomonadales;f__Moraxellaceae;g__Acinetobacter;s__calcoaceticus-pittii
k__Bacteria;p__Proteobacteria;c__Gammaproteobacteria;o__Pseudomonadales;f__Moraxellaceae;g__Acinetobacter;s__ursingii
k__Bacteria;p__Proteobacteria;c__Gammaproteobacteria;o__Pseudomonadales;f__Pseudomonadaceae;g__Pseudomonas;s__aeruginosa
k__Bacteria;p__Proteobacteria;c__Gammaproteobacteria;o__Pseudomonadales;f__Pseudomonadaceae;g__Pseudomonas;s__azotoformans-fluorescens-synxantha
k__Bacteria;p__Proteobacteria;c__Gammaproteobacteria;o__Pseudomonadales;f__Pseudomonadaceae;g__Pseudomonas;s__graminis
k__Bacteria;p__Proteobacteria;c__Gammaproteobacteria;o__Xanthomonadales;f__Xanthomonadaceae;g__Stenotrophomonas;s__maltophilia
